# New Cytoplasmic Virus-Like Elements (VLEs) in the Yeast *Debaryomyces hansenii*

**DOI:** 10.3390/toxins13090615

**Published:** 2021-09-01

**Authors:** Xymena Połomska, Cécile Neuvéglise, Joanna Zyzak, Barbara Żarowska, Serge Casaregola, Zbigniew Lazar

**Affiliations:** 1 Department of Biotechnology and Food Microbiology, Faculty of Biotechnology and Food Science, Wrocław University of Environmental and Life Sciences (WUELS), 50-375 Wroclaw, Poland; barbara.zarowska@upwr.edu.pl (B.Ż.); zbigniew.lazar@upwr.edu.pl (Z.L.); 2 SPO, INRAE, Montpellier SupAgro, Université de Montpellier, 34060 Montpellier, France; cecile.neuveglise@inrae.fr; 3 Department of Microbiology, Laboratory of Microbiome Immunobiology, Ludwik Hirszfeld Institute of Immunology and Experimental Therapy, Polish Academy of Sciences, 53-114 Wroclaw, Poland; j.m.zyzak@gmail.com; 4 INRAE, AgroParisTech, Micalis Institute, CIRM-Levures, Université Paris-Saclay, 78350 Jouy-en-Josas, France; s.casaregola@gmail.com

**Keywords:** yeast, *Debaryomyces hansenii*, virus-like elements (VLEs), linear dsDNA plasmids, killer activity, osmotolerance, killer toxins

## Abstract

Yeasts can have additional genetic information in the form of cytoplasmic linear dsDNA molecules called virus-like elements (VLEs). Some of them encode killer toxins. The aim of this work was to investigate the prevalence of such elements in *D. hansenii* killer yeast deposited in culture collections as well as in strains freshly isolated from blue cheeses. Possible benefits to the host from harboring such VLEs were analyzed. VLEs occurred frequently among fresh *D. hansenii* isolates (15/60 strains), as opposed to strains obtained from culture collections (0/75 strains). Eight new different systems were identified: four composed of two elements and four of three elements. Full sequences of three new VLE systems obtained by NGS revealed extremely high conservation among the largest molecules in these systems except for one ORF, probably encoding a protein resembling immunity determinant to killer toxins of VLE origin in other yeast species. ORFs that could be potentially involved in killer activity due to similarity to genes encoding proteins with domains of chitin-binding/digesting and deoxyribonuclease NucA/NucB activity, could be distinguished in smaller molecules. However, the discovered VLEs were not involved in the biocontrol of *Yarrowia lipolytica* and *Penicillium roqueforti* present in blue cheeses.

## 1. Introduction

In addition to the genetic information in the form of DNA existing in the nucleus, yeast may have extra genetic elements located in the cytoplasm in the form of virus-like particles (VLPs) containing dsRNA as well as circular or linear dsDNA plasmids [1,2]. Linear dsDNA plasmids at present called virus-like elements (VLEs) due to their likely viral origin represent the predominating supplementary genetic material in yeasts. However, the number of discovered VLEs carrying strains is not very large. As a result of analysing over 1800 strains belonging to approximately 600 yeast species, molecules of this type have been found only in 40 strains [1,3,4,5,6,7,8,9,10,11,12,13]. Most of them belong to the Ascomycota phylum (34 strains) and the rest to the Basidiomycota phylum. In the latter, all discovered VLEs containing strains pertain only to one species, *Tausonia pullulans*, formerly *Trichosporon pullulans* [1,14]. In Ascomycota, the largest group (17 strains) belongs to the Debaryomycetaceae family. According to the current taxonomy, these strains belong to the following species: *Babjeviella inositovora* (formerly *Pichia inositovora*), *Millerozyma (Pichia) acaciae*, *Schwanniomyces (Debaryomyces) etchellsii*, *Schwanniomyces (Debaryomyces) occidentalis*, *Schwanniomyces (Debaryomyces) polymorphus*, *Schwanniomyces vanrijiae* var. *yarrowii, Debaryomyces (Wingea) robertsiae*, and *Debaryomyces hansenii* [15,16,17]. Other strains are located within four families: Saccharomycetaceae, Pichiaceae, Phaffomycetaceae, Saccharomycopsidaceae, and Trichomonascaceae [1,3,4,5,9,10,11,12].

VLEs are usually found in pairs in yeast cells. There are also some cases of individual elements or systems consisting of three components [1,8,12]. Except for pPH1 from *Pichia heedii* and pPK1 and pPK2 *from Pichia kluyveri* discovered in mitochondria [1,12], all these elements are located in the cytoplasm [9,12,18,19]. Fragments of VLEs, so-called NUPAVs (nuclear sequences of plasmid and viral origin) can also be found in nuclear chromosomes [20]. However, the expression of nuclear genes that originated from such elements is abolished because of their high A+T content leading to the internal cleavage at poly(A) sites of mRNA and finally transcript fragmentation [21].

The largest elements in VLEs systems are considered autonomous, as they have genes encoding all enzymes necessary for their replication and transcription in cytoplasm regardless of the nuclear DNA [22,23,24,25]. Other molecules (non-autonomous) require the support of the enzymes originating from autonomous elements, e.g., RNA polymerase, helicase, capping enzyme, terminal recognition factor (TRF1), or single-strand DNA binding (SSB) proteins. However, some molecules such as pGKL1 from *Kluyveromyces lactis*, pPin1-3 from *B. inositovora*, pDHL1, and pDH1A from *D. hansenii*, may encode their own B-type DNA polymerase together with the terminal protein (TP), required for their replication. TP, along with SSB proteins and TRF1, is an essential part of the replication initiation complex of VLEs [26]. In some cases, e.g., pGKL1 or pPin1-3, it was also found that genes encoding killer toxins acting as anticodon nucleases (ACNases) and immunity determinants to these killer toxins may be located on non-autonomous elements [21,27,28,29,30].

One of the yeast species in which different VLE systems have been detected is *D. hansenii*, which is quite commonly found in low temperature or low water activity environments such as seawater, glaciers, refrigerated food, non-alcoholic beverages, wine, dairy, and meat products [31,32,33,34]. Although yeasts from this species are generally considered non-pathogenic receiving from EFSA the status of qualified presumption of safety (QPS) and the permission for use in food production [35], they have also been sporadically isolated from humans [36,37]. They occur in large numbers in ripening cheeses where they constitute the major yeast population at the end of the process, despite relatively low proteolytic and lipolytic activities [38]. This is due to their ability to assimilate lactose as well as lactic and citric acids, growing at low temperature and relatively high salt concentrations and, due to the formation of killer toxins at acidic pH [39,40].

It is known that these yeasts, depending on the strain, can secrete various killer toxin proteins. Their common feature is that they hydrolyze β-D-glucan present in the fungal cell walls. Two types of toxins have been identified acting on either (1→6) or (1→3)-β-D-glycosidic bonds. The enzyme of the first type may have different sizes, depending on the strain, ranging from 23 kDa [41] to even 232 kDa [42]. Additionally, one strain can secrete two killer proteins simultaneously [42]. The second type of toxin has been detected by Çorbacı and Uçar [43]. The molecular weight of the protein is 31.5 kDa. All mentioned *D. hansenii* toxins are encoded in the nuclear genome and although *D. hansenii* can also have additional genetic information in the form of VLEs in their cytoplasm, no connection between the killer activity of *D. hansenii* and the possession of VLEs has been found [8,9].

So far, four different plasmid systems have been discovered in representatives of *D. hansenii* species. The first of them, the three-elements system pDHL1/2/3 (8.4/9.2/15.0 kb), was isolated by Gunge et al. [8] from strain TK (CBS 7848) selected from Japanese pickles “Takuan”. The second, detected in strain CBS 770, isolated from the throat of angina patient, consists of two plasmids pDH1A/B of 8.2 and 14.4 kb, respectively [9]. Fukuhara [1] detected two additional strains having such molecules, CBS 1780 (pDH2A/B; 6.5/15 kb) and CBS 4890 (pDH3A/B; 4.7/13 kb). However, the function of all discovered VLEs in *D. hansenii* cells remains unknown.

Previously, we have been investigating the function of different yeast species including *D. hansenii* in the cheese ripening process and the possibility to utilize selected strains to shorten the cheese maturation [44,45,46]. We have found that *D. hansenii* strains originating from the cheese environment express a strong killer activity [39,47,48]. Furthermore, we have developed an industrial technology to produce preparations containing *D. hansenii* killer toxins for plant protection against fungal phytopathogens [49]. The technology has been implemented by Skotan S.A. (Poland) and the product named HanseFlora® is now available in Poland. In the current study, we have expanded our interests to the dissemination of VLEs in *D. hansenii* strains mainly occurring in cheese but also in other ecological niches. We tested strains deposited for years in culture collections as well as freshly isolated from the environment. By analyzing the sequence of three selected new VLE systems, we tried to find/predict a benefit for the host coming from having such elements in the cell.

## 2. Results and Discussion

### 2.1. Frequency of VLEs in D. hansenii Yeast

A set of 135 *D. hansenii* yeast strains was analyzed for the occurrence of VLEs; 75 of them were obtained from culture collections and 60 strains were freshly isolated from the environment (Table 1 and Table 2). Culture collection strains originated mainly from dairy sources but also from ice and seawater, alcoholic and non-alcoholic drinks, meat, insects, and human skin. New isolates were gained from blue-veined cheeses, which are known to be abundant yeast microbiota [40]. To collect a large diversity of samples, eight different types of cheese produced in four European countries were selected. Isolation of yeast strains was performed on a specific medium named YORS and developed particularly for this work. This medium was based on YPD agar (Y) supplemented with oxytetracycline (O), rose bengal (R), and salt (S). Such composition allowed the selective isolation of halophilic yeast such as *D. hansenii* while inhibiting the growth of bacteria, filamentous fungi, and other yeast species present in blue-veined cheeses.

The type strain of *D. hansenii*, CLIB 197^T^, forms colonies of characteristic morphology on YORS agar: pink color, smooth surface, circular shape with pulvinate elevation, and entire margin (Figure S1). Randomly selected strains looking similar to the type strain were chosen for further experiments. All of them were identified as *D. hansenii* by colony PCR with a species-specific pair of primers—DhPADF and DhPADR developed by Wrent et al. [50], giving the product of 402 bp. In each case, a band of approximately 400 bp was obtained (Figure S2). The application of this method significantly reduced the time and cost of the analysis compared to amplification and sequencing of the D1/D2 domain of the large ribosomal subunit [51]. The relatively small size of the PCR product allowed us to carry out the PCR with biomass instead of purified DNA, thus, avoiding the time-consuming nucleic acid isolation required by the aforementioned method but also during the identification of *D. hansenii* by means of intergenic spacer rDNA amplification with subsequent restriction fingerprinting [52,53].

Among yeasts obtained from culture collections, no strain possessing VLEs was detected directly after reviving or after repeated cultivation in a medium containing sodium chloride, which can facilitate the proliferation of cells containing such elements [54]. The procedure of repeated cultures was used for *D. hansenii* CBS 770 and *D. hansenii* CBS 7848. It proved to be effective since no VLEs were observed in DNA preparations isolated from cultures directly after recovery from the freeze-dried preparation, while after six passages in YPD with 12% NaCl VLEs were successfully detected (Figure S3).

At present, it is difficult to find out whether the lack of the cytoplasmic DNA elements in the studied strains was due to their lack in the primary culture or have been caused by isolating these strains on standard yeast media or the following methods of preservation and storage. Strains of CIRM-Levures are stored in liquid nitrogen or in freeze-dried form, while cultures from DBFM are henceforth stored at −80 °C in a glycerol solution. However, for many years they have been stored under paraffin on YM agar slants at a temperature of 4 °C and have been regularly transferred once a year onto a fresh medium. Such a procedure could have led to the gradual loss of plasmids. No experiments about VLEs content have been conducted before culture conservation in both collections, but it should be implemented from now on. Consideration should, therefore, be given to the development of proper storage or revival conditions for strains deposited in culture collections, which could subsequently enhance chances for maintaining cytoplasmic DNA molecules in cells.

It appeared far more efficient to search for VLEs in fresh isolates of *D. hansenii* species obtained directly from cheeses with mould overgrowth. In this case, 25% of them contained such molecules (Table 3). There was high variability in the number of VLEs possessing strains between cheeses; VLEs were observed in half of the cheese samples tested. In most cases, low detection rates in the rest of the trials could be correlated with the small number of analyzed strains (four isolates). In cheeses, where yeasts with VLEs were detected, their frequency ranged from 17% to as much as 50% of isolates, which suggested an important contribution of these elements to yeast cell functioning in such an ecosystem.

**Figure 1:**
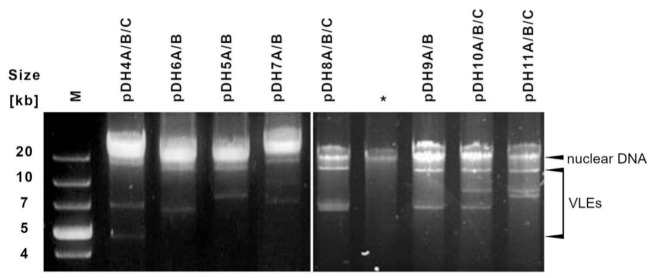
New VLEs systems discovered in *D. hansenii* strains freshly isolated from mould ripened cheeses. Names of yeast strains possessing particular systems are given in Table 3. * Genetic material isolated from strain without VLEs. M. Gene Ruler 1kb Plus DNA Ladder (Thermo Fisher Scientific, Warszawa, Poland).

We also observed that in a given cheese both may occur, strains having the same VLE system, as in the case of pDH4A/B/C in the German Edelpilz cheese, as well as coexisting strains with different sets of such elements, as in strains isolated from the Polish Turek niebieski cheese (Table 3, Figure 1). In the latter, four VLEs possessing strains were detected, i.e., 8e, 8g, 8h, and 8i, and each of them had a different set of such molecules. Based on current data, it cannot be distinguished whether they arise from different ancient rearrangements of an ancestral VLEs system or whether there are undergoing current modifications.

In total, eight different VLEs systems have been distinguished: four binary and four ternary (Figure 1, Table 3). The biggest molecule in each system had a consistent size of 15.1 kb for all tested strains, which is longer than the longest previously known element pSKL from *Lachancea kluyveri* of 14.2 kb [55]. The size variation was observed for the smallest and medium-sized elements. The length of the smallest one ranged from 4.8 to 9.2 kb and the medium one from 7.5 to 10.0 kb.

### 2.2. The Characteristics of Selected VLEs Systems

Three newly discovered sets of VLEs (pDH4A/B/C, pDH5A/B, and pDH6A/B) were selected for next-generation sequencing. They were isolated from strains acquired from cheeses produced in Germany, Poland, and Denmark (strains 4e, 5c, and 7g). The libraries were made from DNA fractions containing a mixture of linear DNA plasmids separated from mitochondrial and nuclear DNA by ultracentrifugation in CsCl-bisbenzimide gradient (Figure S4). This approach was not previously used in the preparation of yeast VLEs for sequencing. So far, labour-intensive purification of individual molecules and their subsequent Sanger-based sequencing has been conducted [24,26,55,56,57,58].

Overlapping reads were assembled into contigs which were compared with GenBank nucleotide database using blastn [59]. It allowed identifying VLE sequences and contaminations with mitochondrial DNA. Such contamination was probably created at the fraction collection stage after separation in the CsCl-bisbenzimide gradient. Due to the small difference in buoyant density between mtDNA and VLEs, both fractions are faintly divided from one another and therefore easy to mix with each other. Nuclear DNA fragments have also been identified among the obtained sequences. Their presence indicates that despite careful operation chromosome fragmentation has not been avoided. Apart from the random fragments of different chromosomes, the sequences of nuclear origin belonged in many cases to the rRNA encoding genes, which are located in many copies in three loci in the genome of *D. hansenii* CBS 767^T^ [60]. In addition, fragments of LTR-retrotransposon Tdh5 have also been identified [61]. Frequent detection of the latter may be associated with the presence of more than 20 copies of this element in the *D. hansenii* genome and subsequent generation of many extrachromosomal DNA copies of this element. While the length of Tdh5 retrotransposons is approximately 5.3 kb, their isolation together with the plasmid fraction was also possible.

Contigs assigned to VLEs constituted almost complete plasmid sequences. In the case of four of them (pDH4C, pDH5B, pDH6A, pDH6B), only the fragment of one terminal inverted repeat (TIR) sequence was missing, while for the other plasmids, sequences at both ends had to be filled by primer walking. Open reading frames were annotated using CLC Sequence Viewer v.8.0.0 (Qiagen Digital Insights). This software was selected based on previously analysed ORF detection accuracy on already known VLEs sequences: pGKL2, pPE1B, pPac1-1, and pSKL. The minimal ORF coding capacity was set to 70 aa as genes encoding 70 aa polypeptides were discovered on pGKL2 and pSKL and proved to be functional [62]. The annotated sequences of pDH4A/B/C, pDH5A/B, and pDH6A/B were deposited in GenBank under the following accession numbers: MF795091/MF795092/MF795093, KX904874/ KX904875, and KX858805/ KX904876, respectively.

**Figure 2:**
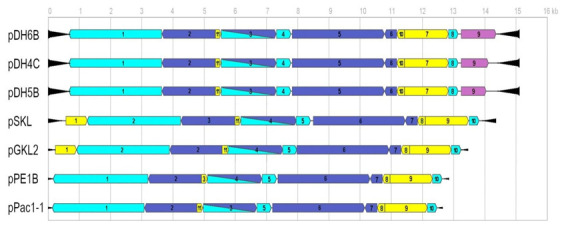
The schematic layout of open reading frames (ORFs) location on newly sequenced autonomous VLEs of *D. han-senii* (pDH4C, pDH5B, pDH6B) and previously known from other yeast species. The characteristics of individual ORFs is described in Table 4 and Table 5. The arrows of light blue color correspond to ORFs encoding enzymes involved in replication, dark blue in transcription, and yellow of unknown function. The products of ORFs colored in purple show some similarities with immunity determinants to VLE killer toxins. The direction of arrows indicates the transcription direction; black triangles represent terminal inverted repeats (TIRs) at the ends of plasmids.

**Figure 3:**
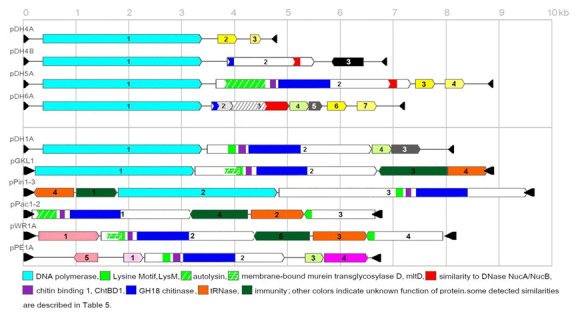
The scheme of open reading frames (ORFs) organization on newly sequenced non-autonomous VLEs of *D. hansenii* (pDH4A/B, pDH5A, pDH6A) and previously known from other yeast species. The characteristics of individual ORFs is described in Table 6 and Table 7. The direction of arrows indicates the transcription direction; black triangles represent terminal inverted repeats (TIRs) at the ends of plasmids. Homologous ORFs or functional domains are marked with one color. DNA fragments encoding full domains are ended with vertical lines from both sides, while partial or with some similarities with arrows.

All new VLEs turned out to be extremely A+T rich (73−76%) and compact in structure, exactly as for previously known plasmids. Their coding capacity was also very high; 75−83% and 88−90% for non-autonomous and autonomous molecules, respectively. At both ends of all analysed VLEs, terminal inverted repeats (TIR) strictly specific to the element type were found (Figure 2 and Figure 3). Those with a length of 715−716 bp detected on autonomous elements turned out to be about 230 bp longer than the longest TIRs previously reported on pSKL. In contrast, TIRs from the smallest VLEs in the systems also proved to be the smallest known (91 bp) and were identical to the 91-first bases of pDH1A TIRs, which are 96 bp in length [58]. In the case of the middle non-autonomous elements, inverted repeats located at the ends were composed of TIR sequences from the small molecules extended for 33 bp.

Each of the newly sequenced VLEs had its own DNA polymerase-encoding gene that enables its replication in the cytoplasm independently of the nucleus using a previously disclosed protein-primed mechanism [reviewed by 30]. The sequence of this gene was closely related to the type of element, highly conserved for autonomous (99−100% identity) and non-autonomous (95−98% id.) molecules, while much more diverse between genes from these two types of VLEs (~ 29% id.).

**Figure 4:**
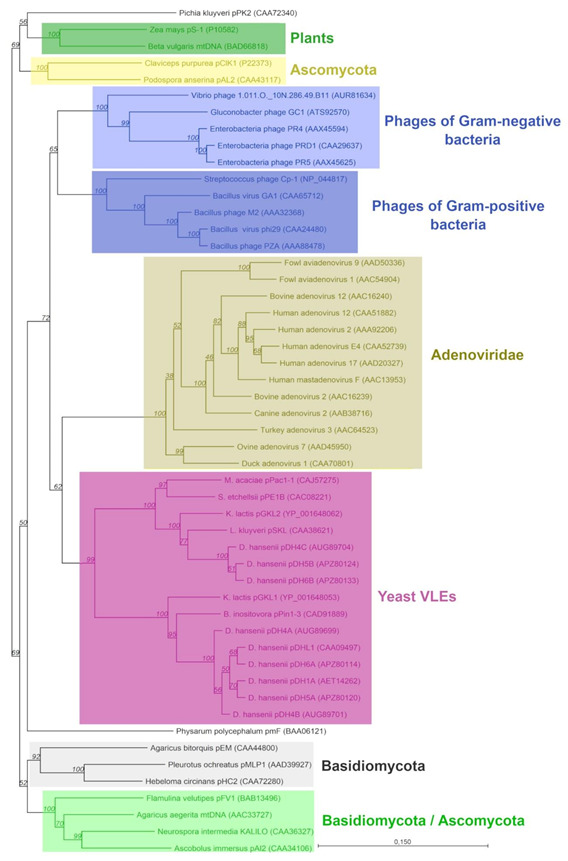
The phylogenetic relationship between B-type DNA polymerases of yeast cytoplasmic VLEs, viruses, and mitochondrial plasmids of different organisms. The phylogram was constructed with the neighbor-joining method using aligned full amino acid sequences of 51 proteins obtained from GenBank and from this study. GenBank accession numbers are given in brackets. The tree topology was verified with a bootstrap method with 1000 replications and the bootstrap percentage was presented on each node. The scale bar shows the number of changes per position.

The confirmation of distinctiveness of these two kinds of DNApol can also be found in the phylogenetic tree illustrating the relationships between various groups of proteins that are representatives of the type B superfamily of DNA polymerases (Figure 4). Similar phylogenetic analyses have been performed before but on a smaller number of sequences [57,58,63,64]. This time, the analysis was expanded due to an increase in the number of available data. Nevertheless, proteins encoded by autonomous and non-autonomous plasmids formed separated groups within the clade of VLEs-origin DNApols. It can be hypothesized that both groups derived from a common ancestor in which the DNApol gene was probably duplicated, then single copies were divided into two linear molecules of different functions and further differentiated. Moreover, our results indicated that VLEs-origin DNA polymerases are closely related to DNApol of adenoviruses and bacteriophages of both Gram-positive and -negative bacteria, which suggests a common origin. The structure of the phylogenetic tree obtained in this work showed that mitochondrial DNApols differentiated earlier from the common progenitor than those of yeast cytoplasmic VLEs, Adenoviridae, and phages. Furthermore, the evolution of plasmid DNApol of mitochondria apparently followed more complicated paths. The basis for this presumption is that the DNApol of mitochondrial linear plasmids of fungi (Ascomycota and Basidiomycota) are arranged in three different clades distant from cytoplasmic and viral B-type polymerases. Therefore, our results confirm the conclusions already reached by Klassen and Meinhardt [64] from the analysis of a smaller pool of sequences.

It seems that genes of DNA polymerases have been pulled into VLEs that were sourced from a different origin. The research of Sýkora et al. [65] and Vopálenský et al. [66], who analyzed the transcriptional mechanism of pGKL from *K. lactis*, showed that RNA polymerase encoded on the autonomous molecules as well as the promoter sequences of all genes lying on both autonomous and non-autonomous molecules probably derived from the protoplast of poxviruses. Furthermore, the DNA polymerases found in viruses of the Poxviridae family do not show homology to the polymerases of VLEs origin.

Sequence analysis of pDH4C, pDH5B, and pDH6B showed their extraordinary conservation, with 99% identity. Therefore, in those molecules, all open reading frames were located precisely in the same orientation and order, and 10/11 of them had the same length, start, and stop codons (Figure 2, Table 4). Individual plasmids differed only in the length of the ORF9 located on the right end. In pDH6B, this ORF was the longest (1104 bp), while in pDH4C and pDH5B, two and one nucleotide insertions, respectively, resulted in the shift of the reading frame and the early appearance of a stop codon, which shortened the ORF length to 861 and 786 bp. A similar genetic organization of core 10 ORFs has already been reported for pGKL2, pPE1B, pPac1-1, and pSKL [22,24,25,55]. However, no homology to DhORF9 was found in these plasmids, although, the additional ORF (ORF1) was found on the left side of pSKL and pGKL2.

The predicted products of all extra ORFs located on VLEs of different species show some similarities to proteins providing immunity to plasmid-encoded killer toxins (Table 5). The putative DhORF9p appears to be most closely related to the immunity determinant of *W. robertsiae* encoded by pWR1A ORF5 (35% identical amino acids on 90% of protein length) and of *M. acaciae* pPac1-2 ORF4 (36% id. aa of 83% of p.l.), which are also closely related to each other (49% id. aa of 97% p.l.). No significant similarity of DhORF9p was found to the zymocin immunity protein encoded by pGKL1 ORF4. The deduced immunity-like proteins of the pSKL and pGKL2 probably belong to a different group of proteins resembling immunity as they have 48% common amino acids on the length of 80% of the protein sequence and they are homologous to the mentioned zymocin resistance protein (25% id./52% p.l.).

**Table 5:** Functional characteristics of autonomous VLEs from *D. hansenii* yeast (GenBank Acc. No.: pDH4C-MF795093, pDH5B-KX904875, pDH6B-KX904876).

ORF	Protein Length [aa]	Predicted Function/Similarity ^1^
1	998	B-type DNA polymerase
2	565	Similarity to putative mRNA capping-enzymes from *M. acaciae* pPac1-1 and *S. etchellsii* pPE1B
3	586	Helicase-DEXDc and HELICc domain containing protein
4	161	Similarity to putative ssDNA binding protein from *M. acaciae* pPac1-1 and *S. etchellsii* pPE1B
5	987	RNA polymerase larger subunit
6	130	Similarity to putative RNA-polymerase subunits from *S. etchellsii* pPE1B (ORF7) and *M. acaciae* pPac1-1 (ORF7)
7	463	Unknown
8	105	DNA-binding protein TRF1 (terminal region recognition factor 1)
9	286/261/367	Similarity to immunity determinants to killer toxins encoded by *M. acaciae* pPac1-2 and *D. robertsiae* pWR1A, fragment of *M. farinosa* chromosome F (XP_004202381)
10	77	Unknown, high similarity to product of ORF8 from: *L. kluyveri* pSKL, *M. acaciae* pPac1-1, *K. lactis* pGKL2, *S. etchellsii* pPE1B
11	62	Unknown, high similarity to products of: *S. etchellsii* pPE1B ORF3 *M. acaciae* pPac1-1 ORF11

^1^ The function of open reading frames was at first predicted based on blastx [67], then in case of no hits Conserved Domains Database [68], as well as pfam database [69], were searched against protein queries for conserved domains.

The function of the immunity-like genes is as yet unknown. However, Schaffrath et al. [70] demonstrated using pGKL2ORF1p as an example that this protein does not guarantee resistance to zymocin. In addition, they also showed that this protein does not participate in the replication processes and maintenance of the VLE system.

*DehaORF9* appears to be intact with a putative upstream control region (UCR) acting as yeast plasmid gene’s promoter [71,72,73]. This sequence contains the ATA TGA key fragment located 38 bp upstream from the start codon. Such sequences (consensus: ATNTGA), called upstream conserved sequences (UCS), usually precede the start codon about 100 bp for autonomous and around 30 bp for non-autonomous plasmids [25,30,70,74,75]. UCS in the promoter sequence is necessary for the correct binding of RNA polymerase of VLEs origin and handling of the transcription of plasmid genes in the cytoplasm.

The sequences of new non-autonomous VLEs were searched for genes, which could be involved in killer toxin production. In all new systems, ORFs containing either a full domain of chitinase activity (GH18) or their fragments were detected (Figure 3, Table 6 and Table 7). Polypeptides with such function are an inseparable part of VLE-encoded killer toxins [27,29]. However, they are also present on cryptic elements which function is still unknown [9,58]. The most interesting ORF containing GH18 is located on pDH5A. The predicted protein encoded by ORF2 contains the full domain of chitinase along with the domain responsible for chitin binding (ChBP1). There is also an incomplete autolysin domain of 1,4-beta-N-acetylmuramyl- hydrolase activity. The autolysin contains elements of the LysM domain that is responsible for binding to peptidoglycan in bacterial cell walls as well as fungal chitin which is structurally similar [76]. LysM domains were also detected in proteins encoded on plasmids pDH1A, pGKL1, pPin1-3, pPac1-2, pWR1A, and pPE1A (Figure 3).

**Table 6:** The characteristics of open reading frames (ORFs) detected on new non-autonomous VLEs of *D. hansenii* yeast: pDH4A (GenBank Acc. No. MF795091), pDH4B (MF795092), pDH5A (KX904874), and pDH6A (KX858805).

VLE	ORF	DNA Strand	ORF Length [bp]	Stop Codon	UCS	UCS Position [bp] ^1^
pDH4A	1	+	3003	TGA	ATG TGA	−25
	2	+	363	TAA	ATA TGA	−27
	3	+	222	TGA	ATG TGA	−26
pDH4B	1	+	3003	TGA	ATG TGA	−25
	2	+	1647	TAA	ATA TGA	−254
	3	−	585	TAG	ATG TGA	−28
pDH5A	1	+	3000	TGA	ATG TGA	−25
	2	+	3681	TAA	ATA TGA	−27
	3	+	363	TAA	ATG TGA	−26
	4	+	369	TGA	ATG TGA	−26
	1	+	3003	TGA	ATG TGA	−27
pDH6A	2	+	396	TAA	ATT TGA	−34
	3	+	1092	TGA	TTA TGA	−66
	4	+	360	TGA	ATG TGA	−24
	5	+	264	TAA	ATA TGA	−40
	6	+	363	TAA	ATG TGA	−26
	7	+	369	TAA	ATG TGA	−26

^1^ described below Table 4.

Additionally, in pDH5AORF2p, a fragment of the deoxyribonuclease domain (DNase NucA/NucB) was also found. Parts of DNase NucA/NucB were also present in other pDH systems, i.e., in ORF2 of pDH4B and in ORF3 of pDH6A. The prediction of protein localization with DeepLoc-1,0 [77] revealed that pDH5AORF2p and pDH6AORF3p can be extracellular proteins. However, only in pDH5AORF2p was the signal peptide of 18 aa detected confirming the possible secretion of this protein outside the cell. Interestingly, in each of the pDH systems, a protein that probably functions in the nucleoplasm (pDH4AORF3p, pDH5AORF4p, and pDHORF7p) was detected. These proteins are almost identical and highly homologous to the hypothetically cytoplasmic pDH4AORF2p, pDH5AORF3p, and pDH6AORF6p proteins. It could be assumed that these proteins may be an element of the host’s genetic material defense against the killer activity of DNases.

**Table 7:** The analysis of predicted proteins based on open reading frame (ORF) sequences detected on new non-autonomous VLEs of *D. hansenii* yeast: pDH4A (GenBank Acc. No. MF795091), pDH4B (MF795092), pDH5A (KX904874), and pDH6A (KX858805).

VLE	ORF	Protein Length [aa]	Subcellular Localization Prediction ^1^	Predicted Function or Functional Domains/Similarity ^2^
pDH4A	1	1000	Cytoplasm	B-type DNA polymerase
	2	120	Cytoplasm	Unknown, identical to pDH5A ORF3p and pDH6A ORF6p, high homology to pDH4A ORF3p, pDH5A ORF4p, pDH6A ORF7p and *B. inositovora* XP_018982205
	3	73	Nucleoplasm	Unknown, identical to the part of pDH5A ORF4p and pDH6A ORF7p
pDH4B	1	1000	Cytoplasm	B-type DNA polymerase
	2	548	Peroxisome membrane	Similarity to GH18 chitinase-like superfamily proteins, fragment of DNase NucA/NucB
	3	194	Cytoplasm	Unknown, some similarities to ORF1p from autonomous plasmids pSKL and pGKL1
pDH5A	1	999	Cytoplasm	B-type DNA polymerase
	2	1226 ^3^	Extracellular	Fragment of autolysin domain, full chitin binding 1 domain (ChtBD1), full GH18 chitinase-like domain, fragment of deoxyribonuclease NucA/NucB domain
	3	120	Cytoplasm	as described for pDH4A ORF2p
	4	122	Nucleoplasm	Unknown, identical to pDH6A ORF7p, high homology to pDH4A ORF2p, pDH5A ORF3p and pDH6A ORF6p
	1	1000	Cytoplasm	B-type DNA polymerase
pDH6A	2	131	Mitochondrion matrix	Contains fragment of GH18 chitinase domain
	3	363	Extracellular	Contains fragment of DNase NucA/NucB
	4	119	Cytoplasm	Unknown, homology to proteins of unknown function of *D. hansenii* CBS 767^T^, pDH1A ORF4p and to the part of pPE1A ORF3p
	5	87	Extracellular	Unknown, homology to the part of pDH1A ORF3p
	6	120	Cytoplasm	As described for pDH4A ORF2p
	7	122	Nucleoplasm	As described for pDH5A ORF4p

^1^ Subcellular localization of hypothetical proteins was predicted with DeepLoc-1 [77]. ^2^ The function of open reading frames was predicted as described under Table 3. ^3^ Protein with detected signal peptide.

However, these assumptions need to be confirmed in laboratory experiments. DNase activity has not yet been identified in any protein derived from linear plasmids, including killer ones. However, PaT and zymocin killer toxins indirectly affect DNA integrity in *M. acaciae* and *K. lactis*. These toxins cleave selected tRNAs, which leads to damages in the DNA repair systems and accumulation of untidy mutations [29,78,79,80].

The pDH5AORF2p protein may undergo modifications with KEX2 protease because five motifs recognizable by this enzyme can be found in its sequence: one in the autolysin domain, two in the chitinolytic domain, and two before the fragment of the putative DNase. The post-translational modifications by KEX1/KEX2 proteases are necessary for the secretion of active killer toxins encoded both chromosomally and by VLEs (for a review, see [81]). Further, ORF2p secondary structure analysis with Phobius [82] showed that probably the fragment involved in chitin-binding and its cleavage is localized on the outer side of the cell membrane of the target yeast or mould cell, then, there is one transmembrane region followed by the element of the DNA digesting activity located on the cytoplasmic side.

In the case of the remaining proteins potentially associated with killer activity (pDH4BORF2p, pDH6AORF2-5p), none of them have a signal peptide, the presence of which would suggest the possibility of extracellular secretion. Only pDH4BORF2p has a 31-amino acid transmembrane region located between the fragments identified by the Phobius software [82] as cytoplasmic and non-cytoplasmic. The transmembrane region (beta subunit) is present, e.g., in zymocin, and is probably responsible for the transport of the active gamma subunit to the cytoplasm of the attacked cell [30].

Some ORFs on the non-autonomous VLEs are not conserved in the other *D. hansenii* systems. An example is ORF3 from pDH4B, which has not been detected in other *D. hansenii* VLEs. However, ORF3p showed homology to the immunity-like protein of ORF1 from the autonomous plasmids pSKL and pGKL2. Their function is still unknown but blastp search showed some similarities with alpha-mannosyltransferases, which may suggest that they could be involved in protein glycosylation processes.

### 2.3. The Relationship between New VLEs and Killer Activity

Considering the above data, we decided to check the relationship between new VLEs and killer activity in practice. For that purpose, selected yeasts possessing VLEs were cultured for six weeks in media without selection pressure (without NaCl) and at an elevated temperature (30 °C). Similar conditions caused the loss of the pDHL1/2/3 system in cells of *D. hansenii* CBS 7848 [8]. The lack of VLEs was first confirmed by electrophoretic separation of total DNA isolated from yeast cells, and then, by polymerase chain reaction (Figure S5). Gunge et al. [8] have shown that cells can gradually lose their VLEs from a system, eventually losing their autonomous molecules. Therefore, primers enabling the amplification of a 963 bp DNA polymerase fragment from the autonomous plasmids were designed for PCR reactions. As it is shown in Figure S5, the applied procedure of treatment enabled the complete removal of VLEs from *D. hansenii* cells.

After confirmation of the VLEs’ lack, a cross test was performed with *Y. lipolytica* PII6a yeast and *P. roqueforti* PR1 mould used as sensitive organisms (Figures S6–S9). Both species coexist with *D. hansenii* in blue-veined cheeses and could be the target of their VLE-encoded toxins due to the presence of chitin in their cell wall [83,84]. No killer activity was observed at neutral pH, which is optimal for VLE-origin killer toxins, zymocin, and PaT [30,85]. The test was performed at 14 °C, which is used for blue-veined cheese ripening as well as at 28 °C which is the standard temperature for yeast cultivation. In addition, the loss of immunity of *D. hansenii* strains to its own toxin due to the loss of linear plasmids was also not observed, neither at pH 4.6 nor 7 (Figure S10). The killer effect of the studied strains on *Y. lipolytica* and *P. roqueforti* was observed at pH 4.6, which is optimal for killer toxin activity of *D. hansenii* strains isolated from cheese [39,42,86]. However, no difference in lethal activity between VLEs possessing and cured of them isolates could be noted (Figures S11 and S12). Thus, the antifungal activity of the yeast *D. hansenii* is not encoded within VLEs. It is in accordance with results obtained previously by Gunge et al. [8] for the pDHL1/2/3 system.

### 2.4. The Relationship between Plasmids and Halotolerance

Bearing in mind that the function of linear cytoplasmic genomic elements of *D. hansenii* remains cryptic, it was also examined if their presence increases the yeast resistance to a high concentration of sodium chloride. Although sustaining VLEs in *D. hansenii* cells may be dependent on the presence of salt in the environment [54], there was no change in growth profiles of yeast deprived of such elements in comparison with strains possessing them in YPD medium containing NaCl in the range of 0 to 24% (for an example see Figure S13). Another possibility for *D. hansenii* to maintain VLEs is its putative toxicity against lactic acid bacteria present in cheese. This hypothesis remains to be tested.

## 3. Conclusions

Although it is known that VLEs are the most widespread type of additional genetic information in yeast, we still do not know everything about them. This is due, among other things, to the fact that until now sequences of only three complete VLE systems and four individual linear dsDNA molecules have been deposited and analyzed. In this work, the available data were expanded to include sequences of the full three new systems from one yeast species—*D. hansenii*, including, for the first time, a system consisting of three elements. These plasmids, apart from the genes associated with their own replication and transcription, have ORFs resembling genes of killer activity and resistance. However, our experimental studies showed no relationship between killer activity and the presence of VLEs in host cells and, therefore, the function of VLE-encoded proteins is still not deciphered. Further analyses are needed to check the conditions under which VLE-derived genes are expressed and correlate them with organisms that may be targeted for potential VLE-encoded toxins. It would be also interesting to isolate *Debaryomyces* strains harboring VLEs from different environments than dairy products. The linear plasmid purification procedure followed by NGS used in this work can be utilized for rapid sequencing of similar systems in other organisms and can contribute to a better understanding of their functions.

## 4. Materials and Methods

### 4.1. Microorganisms

The presence of VLEs was studied in the pool of culture collection *D. hansenii* strains originated from Department of Biotechnology and Food Microbiology (DBFM) deposit, WUELS, Poland and from CIRM-Levures, INRA, France (http://www6.inra.fr/cirm/Levures) as well as of new isolates from different blue-veined cheeses produced in several European countries (Table 1 and Table 2). The following strains were used as references for their identification: *D. hansenii* CLIB 197^T^ (=CBS 767^T^), *D. hansenii* CLIB 907 (formerly *C. famata* var. *famata* CBS 1795^T^), *D. hansenii* CBS 7848, *D. hansenii* CBS 770, *D. fabryi* CLIB 422^T^ (=CBS 789^T^), *D. subglobosus* CLIB 908^T^ (=CBS 1796^T^). *Yarrowia lipolytica* PII6a obtained from DBFM, and *Penicillium roqueforti* PR1 (CHR Hansen) served as sensitive cultures for antifungal activity tests. All yeasts used in this study were stored at 4 °C on YPD agar slants (yeast extract 1%, peptone 2%, glucose 2%, agar 1.5%) supplemented with 3% NaCl, except *Y. lipolytica* and *P. roqueforti*, which were kept on YPD agar without salt under the same conditions.

### 4.2. New Yeast Strain Isolation

Yeast isolation was performed on YORS agar (yeast extract 10 g L^−1^, peptone 20 g L^−1^, glucose 20 g L^−1^, oxytetracycline 0.1 g L^−1^, rose bengal 0.033 g L^−1^, NaCl 120 g L^−1^, agar 15 g L^−1^). The medium without oxytetracycline was sterilized at 121 °C for 20 min. After cooling to approximately 90 °C, sterilely weighed oxytetracycline was added to the medium and quickly mixed until dissolved. Cheese samples of 5 g were homogenized in 45 mL of physiological salt solution (Stomacher Lab Blender, Seward, Worthing, UK) and diluted in the same liquid. Decimal dilutions were spread onto the surface of YORS agar. Colonies were taken from plates after 5 days incubation at 25 °C and streaked onto YPD agar plates with 3% NaCl.

### 4.3. Yeast Isolate Identification

As we wanted to analyze only *D. hansenii* strains, new yeast isolates were initially examined by colony PCR with *D. hansenii* species-specific pair of primers DhPadF/DhPadR developed by Wrent et al. [50] under their conditions except for the initial denaturation step, which was prolonged from 5 to 10 min. The reaction was performed with a small portion of 24 hour-old biomass taken from a plate with a 10 µL tip and mixed with 10 µL of 2 × Taq Nova Red Master Mix (Blirt-DNA-Gdansk, Gdansk, Poland), 0.4 µL of 10 µM each primer, and 9.2 µL of distilled water. The product was visualized with ethidium bromide after electrophoresis in 1.2% agarose gel at 120 V for 40 min.

### 4.4. Plasmid Detection

The VLEs presence in cells of new yeast strains was studied immediately after isolation, while for culture collection strains, it was carried out twice: first directly after reviving and then after four propagations in YPD medium with gradually increased NaCl concentration in the range of 0−9%. Each culture was shaken at 25 °C at 160 rpm (G10 Gyrotory Shaker, New Brunswick Scientific Co., Edison, NJ, USA) and after 7 days 1 mL was transferred to a new medium. The last culture was made in YPD containing 12% of NaCl and took 24 h. Then, the biomass was centrifuged at 6000 rpm (Sigma 16−32K), washed with distilled water, and used for DNA isolation.

The total DNA was obtained with GeneMATRIX Bacterial and Yeast Genomic DNA Purification Kit (Eur_x_, Gdansk, Poland) according to the manufacturer’s protocol. Plasmids were separated from nuclear and mitochondrial DNA in 0.8% agarose gel with 1×TAE buffer at 90 V for 1.5 h and visualized with ethidium bromide.

VLEs for NGS sequencing were isolated according to an adapted protocol of Querol et al. [87]. Briefly, cells were harvested, lysed with zymolyase 100T (ICN Biochemicals, Aurora Ohio USA), and treated with SDS. Total DNA was precipitated within ethanol and resuspended in TE buffer. CsCl (1 g mL^−1^) and 10 µL mL^−1^ of 10 mg mL^−1^ stock solution of bisbenzimide (Hoechst; Thermo Fisher Scientific Poland, Warszawa, Poland) were added to total DNA. Then, the plasmid fraction along with mitochondrial DNA was separated by ultracentrifugation (Beckman L7-55 Ultracentrifuge, Beckman Coulter, Indianapolis, IND, USA) after 60 h at 50,000 rpm at 20 °C. The fraction with plasmid DNA was purified four times with isoamyl alcohol buffer made of 100 mL of isoamyl alcohol, 1 mL Tris-HCl 1 M pH 8, 20 g CsCl, and 20 mL water. DNA was precipitated with Na-acetate and isopropanol and finally resuspended in 50 µL of TE buffer.

### 4.5. Sequencing, Assembly and Annotation

VLE containing fractions were used for the construction of libraries, which were sequenced with Illumina MiSeq at Genome S.A. (Warszawa, Poland). The plasmid libraries were run 250 cycles giving paired-end reads of 2 × 250 bp. Raw Illumina reads were trimmed and assembled using CLC Genomic Workbench v. 8.0.0 (QIAGEN Digital Insights). The obtained contigs were blasted against the *D. hansenii* nuclear and mitochondrial genome to exclude contigs not belonging to VLEs [59]. Missing fragments were filled with the primer walking method. Obtained full sequences of linear plasmids were deposited in GenBank under the following accession numbers: pDH4A/B/C- MF795091/MF795092/MF795093, pDH5A/B-KX904874/KX904875, and pDH6A/B-KX858805/ KX904876.

Gene annotation on VLEs was performed with CLC Sequence Viewer (QIAGEN Digital Insights). The function of open reading frames was at first predicted by sequence similarity on the basis of blastx [67]. Then, in case of no hits, the Conserved Domains Database [68], as well as the Pfam database [69], were used to search for conserved domains. Protein secondary structure and subcellular localization were analyzed with Phobius [82] and DeepLoc-1.0 [77].

### 4.6. Phylogenetic Analysis

Amino acid sequences of DNA polymerases were aligned in CLC sequence viewer v.8.0.0 (QIAGEN Digital Insights). The same software was used for the construction of the phylogenetic tree based on the neighbor-joining method with Jukes-Cantor protein distance measure after 1000 bootstrap replications.

### 4.7. VLE Curing

To force the yeast cells to lose linear cytoplasmic DNA, strains were cultivated on YPD medium without NaCl at 30 °C, with shaking at 160 rpm for 7 days and transferring 1 mL into a fresh medium. Such procedure was repeated 8 times. Then, the total DNA was isolated and inspected on agarose electrophoresis as described previously. Furthermore, the isolated DNA was used as a template for PCR reaction with primers designed with Primer3Plus [88] specific for the DNA polymerase gene from autonomous *D. hansenii* VLEs: pCpolDNAF1 (5′ TTG GTG CGA TAT AGA TGG AAA A 3′) and pCpolDNAR1 (5′ TGC TTT TTA AAC CAG CTC CA 3′). The reaction mixture was: 0.4 µL DNA mixed with 10 µL of 2 × Taq Nova Red Master Mix (Blirt-DNA-Gdansk), 0.4 µL of 10 µM each primer, and 8.8 µL of distilled water. The PCR reaction was performed in a Personal Thermocycler (Biometra, Göttingen, Germany) under the following conditions: initial denaturation at 95 °C for 5 min; 35 cycles of denaturation at 94 °C for 1 min, primers annealing at 49 °C for 30s, extension at 72 °C for 1 min; and final elongation at 72 °C for 10 min. The product was visualized with ethidium bromide after electrophoresis in 1.2% agarose gel at 120 V for 40 min.

### 4.8. Antifungal Activity Test

The connection between antifungal activity and the presence of VLEs was tested according to the method described by Woods and Bevan [89]. Strains with such elements and cured of them were streaked in a thick line on a lawn of a sensitive *Y. lipolytica* PII6a strain (5 × 10^5^ cells mL^−1^ of YPD-MB agar pH4.6 or pH 7.0 containing 2.5% NaCl) or *P. roqueforti* PR1 (1 × 10^4^ cells per plate of PDA + 2.5% NaCl pH4.6 and 7.0) and cultivated at 14 °C and 28 °C for 72 h. An analogous experiment was performed with *D. hansenii* strains possessing VLEs streaked on a lawn of *D. hansenii* cured of them. The experiment was performed on YMB pH 7.0 with 2.5% NaCl. The streaked strain was considered a killer yeast if the line of the biomass was surrounded by a clear growth inhibition zone of the sensitive strain. In the case of *P. roqueforti*, changes in sporulation were also inspected. All experiments were run in four replicates.

### 4.9. Halotolerance Test

Strains possessing virus-like elements and cured of them were cultivated on YPD medium with a concentration of NaCl in the range of 0−24%. Cultures were performed in an Automated Microbiology Workstation Bioscreen C (Labsystems Oy, Helsinki, Finland) in a total volume of 350 L in five replications at 28 °C for 96 h with continuous agitation. The BioLink software (Labsystems Oy, Helsinki, Finland) was applied to plot growth curves from optical density measurements performed with a wide band filter (420−580 nm).

## Figures and Tables

**Table 1:** *D. hansenii* strains obtained from culture collections used in the study.

Culture Collection	Strain Designation	Source of Isolation		
DBFM ^1^	AI1a, AI4a, AI4b, AI4c, AI6c, AII2c, AII3b, AII4a,	Dairy products		Rokpol cheese
	AII4b, AII4b-1, AII4c, BI6b, EI2b, EI3a, EI4a, EII2a,			
	EII3c, MI1a, MI1a-1, MI1a-2, MI2a, MI4a, MI5b,			
	MI6b, MI7a, OI1a, OI1b, OI5c, OII1c, OII3a, PI1a,			
	PI4a, PI5b, PII3a			
	DRIP1b, DRIP2c, DRIIP1b,			Dorblu cheese
	DRIIIP3c, DRIW4c, DRIIW3b,			
	DRIIW4c, DRIIIW6a, KI2a			
	DNIP4b, DNIIP2b			Danablu cheese
CIRM-Levures ^2^	CLIB 236	Dairy products and environment		Roncal cheese
	CLIB 380, 594			Goat’s cheese
	CLIB 607			Camembert
	CLIB 608			Air
	CLIB 609, 684			Milk
	CLIB 611			Dairy brine
	CLIB 613			Forage
	CLIB 622			Saint Nectaire
	CLIB 920			Cheese curd
	CLIB 539, 1302, 1465, 1277, 1298, 1301	Ice, glacial, and seawater		
	CLIB 543, 944, 1086, 1389	Beverages		
	CLIB 545, 907	Human		
	CLIB 542, 1295	Meat products		
	CLIB 1142, 1143	Insects		
	CLIB 195, 1144	Unknown		
	CLIB 1296	Salted pickles		

^1^ DBFM: Department of Biotechnology and Food Microbiology culture collection, Wroclaw University of Environmental and Life Sciences, Wroclaw, Poland. ^2^ CIRM-Levures: Centre International de Ressources Microbiennes-Levures, INRA, Jouy-en-Josas, France.

**Table 2:** The origin of new *D. hansenii* isolates.

Strain	Cheese Type	Country
1 (a-d)	Fourme d’Ambert	France
2 (a-d)	Bleu d’Auvergne	France
3 (a-d)	Rokpol Lazur	Poland
4 (a-l)	Edelpilz	Germany
5 (a-f)	Rokpol KG	Poland
6 (a-j)	Dorblu	Germany
7 (a-j)	Bloose	Denmark
8 (a-j)	Turek niebieski	Poland

**Table 3:** Size of new VLEs systems discovered in *D. hansenii* yeast strains.

System	Strains	VLE Size
pDH4A/B/C	4a, 4c, 4d, 4e, 4f, 4i	4.8; 6.9; 15.1
pDH5A/B	5c	8.9; 15.1
pDH6A/B	7f, 7g, 7i	7.2; 15.1
pDH7A/B	7j	8.4; 15.1
pDH8A/B/C	8e	7.4; 8.0; 15.1
pDH9A/B	8g	7.6; 15.1
pDH10A/B/C	8h	7.6; 10.0; 15.1
pDH11A/B/C	8i	9.2; 10.0; 15.1

**Table 4:** Characteristics of open reading frames (ORFs) detected on new autonomous VLEs from *D. hansenii* yeast (GenBank Acc. No.: pDH4C-MF795093, pDH5B-KX904875, pDH6B-KX904876).

ORF	DNA Strand	ORF Length [bp]	ORF Identity [%]	Stop Codon	UCS	UCS Position ^1^ [bp]
1	−	2997	100	TGA	ATATGA	−34
2	−	1698	100	TAA	ATATGA	−52
3	+	1761	99 ^2^	TAA	ATCTGA	−25
4	+	486	100	TGA	ATGTGA	−108
5	+	2964	99 ^3^	TAA	ATGTGA	−50
6	−	393	100	TAA	ATGTGA	−26
7	+	1392	100	TAA	ATTTGA	−43
8	+	318	100	TAA	ATTTGA	−8
9	+	861/786/1104	100 ^4^	TAA	ATATGA	−38
10	−	234	100	TAA	ATTTGA	−34
11	+	186	100	TAA	ATTTGA	−95

^1^ The distance of UCS (upstream conserved sequence) first base from the start codon, ^2^ 1760/1761 identities, ^3^ 2963/2964, ^4^ 100% identities on the first 785 bp of ORF9.

## Data Availability

The sequences of pDH4A/B/C, pDH5A/B and pDH6A/B are available in GenBank (https://www.ncbi.nlm.nih.gov/) under the following accession numbers: MF795091/MF795092/MF795093, KX904874/ KX904875 and KX858805/ KX904876, respectively.

## Supplementary Materials

The following are available online at https://www.mdpi.com/article/10.3390/toxins13090615/s1, Figure S1: The morphology of *D. hansenii* CLIB 197 (CBS 767) type strain colonies on YORS agar medium after 7 days of incubation at 25 °C. Figure S2: The products of colony PCR with *D. hansenii* species-specific pair of primers DhPadF and DhPadR: 1. GeneRuler 100bp Plus (Thermo Fisher Scientific, USA), 2. reagent control, 3. *D. hansenii* CLIB 197^T^ (CBS 767^T^), 4. *D. hansenii* CLIB 907 (CBS 1795), 5. *D. hansenii* 5c, 6. *D. hansenii* 7g, 7. *D. fabryi* CLIB 422^T^ (CBS 789^T^), 8. *D. subglobosus* CLIB 908^T^ (CBS 1796^T^). Figure S3: Comparison of VLEs isolation from yeast biomass obtained after 24h culture in YPD broth inoculated with freeze-dried cells from the collection (2,6) and from biomass gained after six passages in YPD broth with 12% NaCl (4,8). 2,4. *D. hansenii* CBS 7848; 6,8. *D. hansenii* CBS 770. 1,3,5,7. GeneRuler 1kb DNA Ladder (Thermo Fisher Scientific). Figure S4: The separation of virus like elements (VLEs) containing fraction from nuclear and mitochondrial DNA by centrifugation in CsCl-bisbenzimide gradient. Figure S5: Confirmation of *D. hansenii* cell curing of VLEs using PCR with primers specific for autonomous cytoplasmic DNA elements. Lines 1 and 25: GeneRuler 100 bp Plus (ThermoFisher Scientific). Lines 2-10: PCR with DNA isolated from strains before curing, 2: 4e, 3: 5c, 4: 7g, 5: 7j, 6: 8e, 7: 8g, 8: 8h, 9: 8i, 10: CBS 767, 11-12: CBS 7848 before and after curing, 13-14: CBS 770 before and after curing. Lines 15-22: PCR with DNA from VLE-cured strains, 15: 4e, 16: 5c, 17: 7g, 18: 7j, 19: 8e, 20: 8g, 21: 8h, 22: 8i, 23: PCR with purified pDH4C, 24: reagent control. Figure S6: Killer activity test of *D. hansenii* strains before (horizontal streaks) and after (vertical streaks) curing of VLEs against sensitive *Y. lipolytica* PII6a yeast strain (in lawn) on YPG agar pH 7.0 with 2.5% NaCl at 14^o^C. (A) 4e, (B) 5c, (C) 7g, (D) 8h, (E) CBS 7848, (F) CBS 770. Figure S7: Killer activity test of *D. hansenii* strains before (horizontal streaks) and after (vertical streaks) curing of VLEs against sensitive *Y. lipolytica* PII6a yeast strain (in lawn) on YPG agar with 2.5% NaCl pH 7.0 at 28 °C. (A) 4e, (B) 5c, (C) 7g, (D) 8h, (E) CBS 7848, (F) CBS 770. Figure S8: Killer activity test of *D. hansenii* strains before (horizontal streaks) and after (vertical streaks) curing of VLEs against sensitive *P. roqueforti* PR1 (in lawn) on PDA with 2.5% NaCl pH 7.0 at 14 °C. (A) 4e, (B) 5c, (C) 7g, (D) 8h, (E) CBS 7848, (F) CBS 770. Figure S9: Killer activity test of *D. hansenii* strains before (horizontal streaks) and after (vertical streaks) curing of VLEs against sensitive *P. roqueforti* PR1 (in lawn) on PDA with 2.5% NaCl pH 7.0 at 28 °C. (A) 4e, (B) 5c, (C) 7g, (D) 8h, (E) CBS 7848, (F) CBS 770. Figure S10: Killer activity test of *D. hansenii* strains before (streaks) and after (in lawn) curing of VLEs on YMB pH 7.0 with 2.5% NaCl at 14 °C. (A) 4e, (B) 5c, (C) 7g, (D) 8h. The same results were obtained on YMB pH 4.6 with 2.5% NaCl at 14 °C. Figure S11: Killer activity of *D. hansenii* strains before (horizontal streaks) and after (vertical streaks) curing of VLEs against sensitive *Y. lipolytica* PII6a yeast strain (in lawn, dead cells colored in blue) on YMB pH 4.6 with 2.5% NaCl at 14^o^C for 72h. (A) 4e, (B) 5c, (C) 7g, (D) 8h. Figure S12: Antifungal activity of *D. hansenii* strains before (vertical streaks) and after (horizontal streaks) curing of linear plasmids against sensitive *P. roqueforti* PR1 (in lawn) on PDA + 2.5% NaCl pH 4.6 at 14 °C. (A) 4e left and up, 5c down and right, (B) 7g left and down, CBS 7848 up and right, (C) 8h left and up, CBS 770, (D) control plate. Figure S13: The growth of *D. hansenii* 5c strain before (red line) and after (yellow line) VLEs curing in YPD medium with different NaCl concentration. Cultures performed in BioscreenC plotting OD value using wide band filter of range 420–580 nm.
