# Supplementary material for: New Cytoplasmic Virus-Like Elements (VLEs) in the Yeast Debaryomyces hansenii
Source: Toxins (Basel). 2021 Sep 1;13(9):615. doi: 10.3390/toxins13090615 (PMC8472843; doi:10.3390/toxins13090615)
Supplement: Supplementary file 1 [file toxins-13-00615-s001.zip › toxins-1328054-supplementary.pdf]

toxins

Supplementary Materials: New Cytoplasmic Virus-Like Elements (VLEs) in the Yeast *Debaryomyces hansenii*

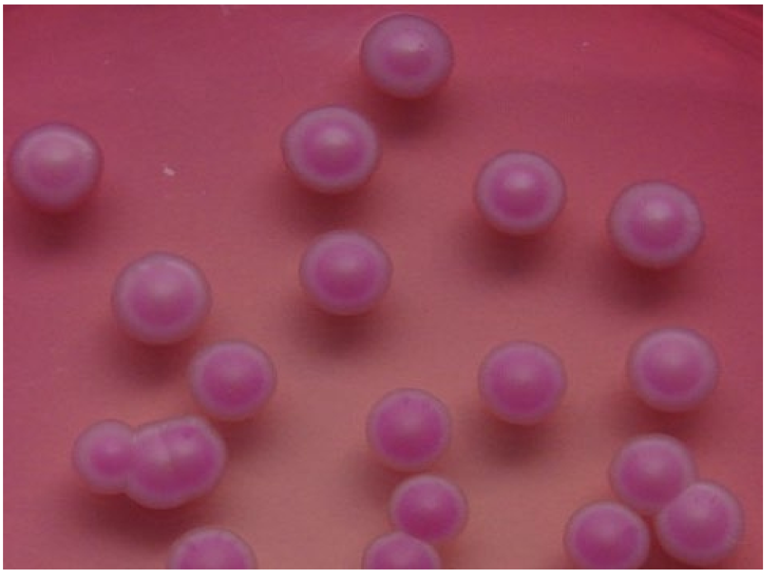

Figure S1. The morphology of *D. hansenii* CLIB 197 (CBS 767) type strain colonies on YORS agar medium after 7 days of incubation at 25 °C.

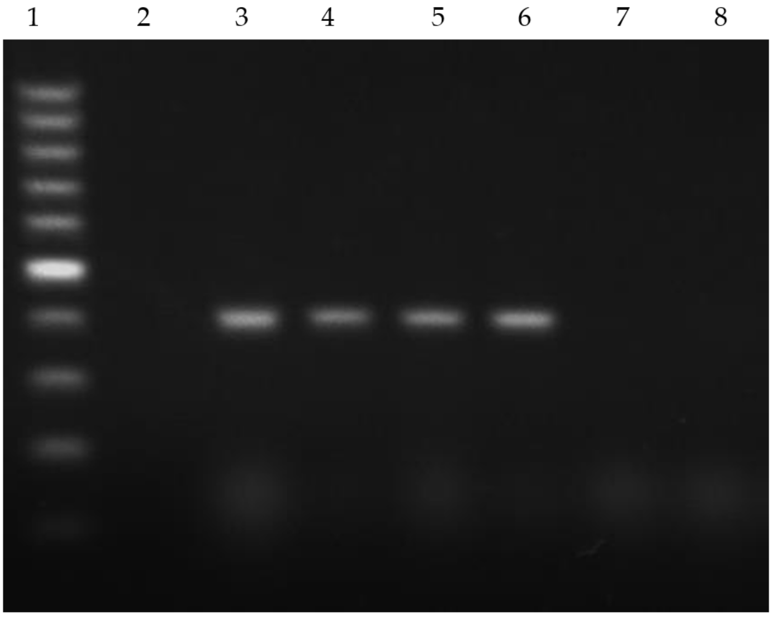

Figure S2. The products of colony PCR with *D. hansenii* species-specific pair of primers DhPadF and DhPadR: 1. GeneRuler 100bp Plus (Thermo Fisher Scientific, USA), 2. reagent control, 3. *D. hansenii* CLIB 197^T (CBS 767^T), 4. *D. hansenii* CLIB 907 (CBS 1795), 5. *D. hansenii* 5c, 6. *D. hansenii* 7g, 7. *D. fabryi* CLIB 422^T (CBS 789^T), 8. *D. subglobosus* CLIB 908^T (CBS 1796^T).

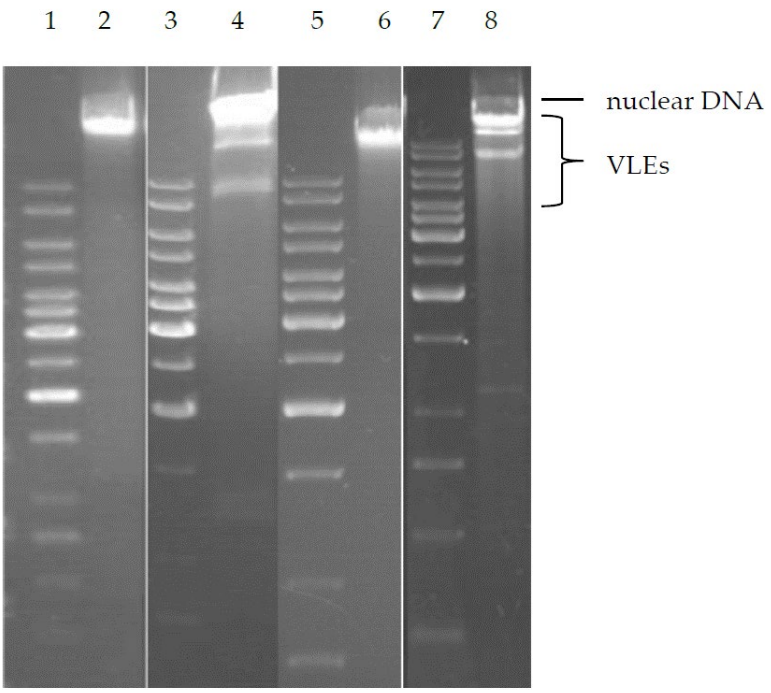

Figure S3. Comparison of VLEs isolation from yeast biomass obtained after 24h culture in YPD broth inoculated with freeze-dried cells from the collection (2,6) and from biomass gained after six passages in YPD broth with 12% NaCl (4,8). 2,4. *D. hansenii* CBS 7848; 6,8. *D. hansenii* CBS 770. 1,3,5,7. GeneRuler 1kb DNA Ladder (Thermo Fisher Scientific).

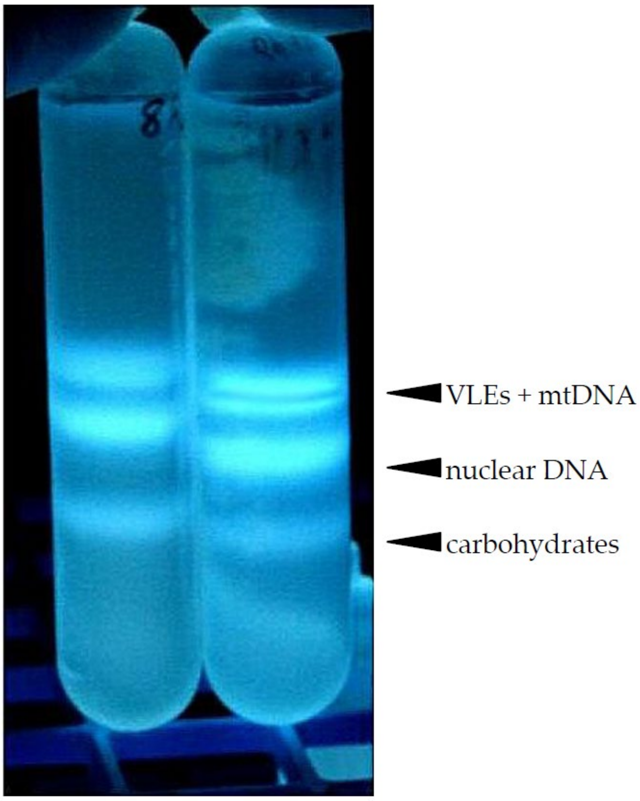

Figure S4. The separation of virus like elements (VLEs) containing fraction from nuclear and mitochondrial DNA by centrifugation in CsCl-bisbenzimide gradient.

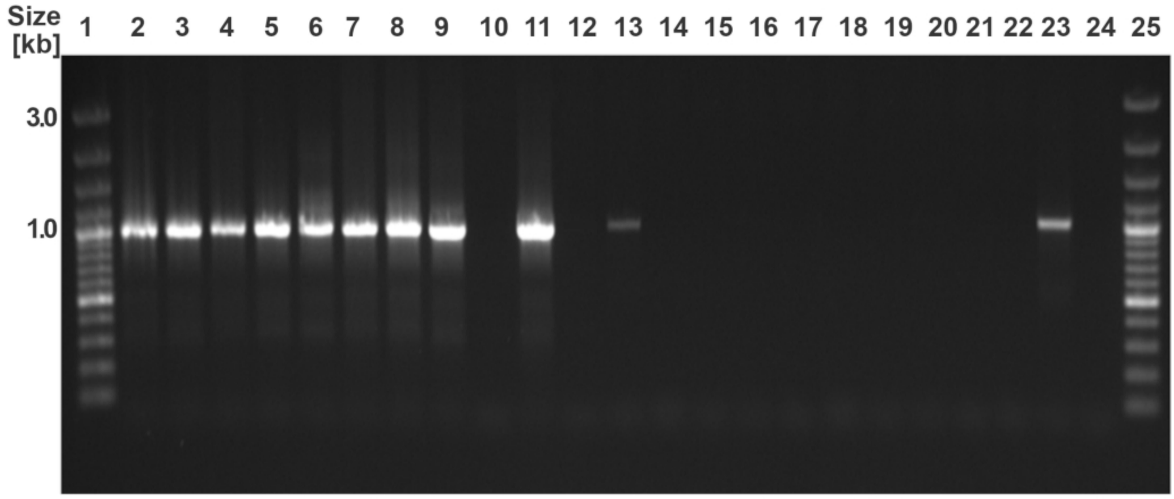

Figure S5. Confirmation of *D. hansenii* cell curing of VLEs using PCR with primers specific for autonomous cytoplasmic DNA elements. Lines 1 and 25: GeneRuler 100 bp Plus (ThermoFisher Scientific). Lines 2-10: PCR with DNA isolated from strains before curing, 2: 4e, 3: 5c, 4: 7g, 5: 7j, 6: 8e, 7: 8g, 8: 8h, 9: 8i, 10: CBS 767, 11-12: CBS 7848 before and after curing, 13-14: CBS 770 before and after curing. Lines 15-22: PCR with DNA from VLE-cured strains, 15: 4e, 16: 5c, 17: 7g, 18: 7j, 19: 8e, 20: 8g, 21: 8h, 22: 8i, 23: PCR with purified pDH4C, 24: reagent control.

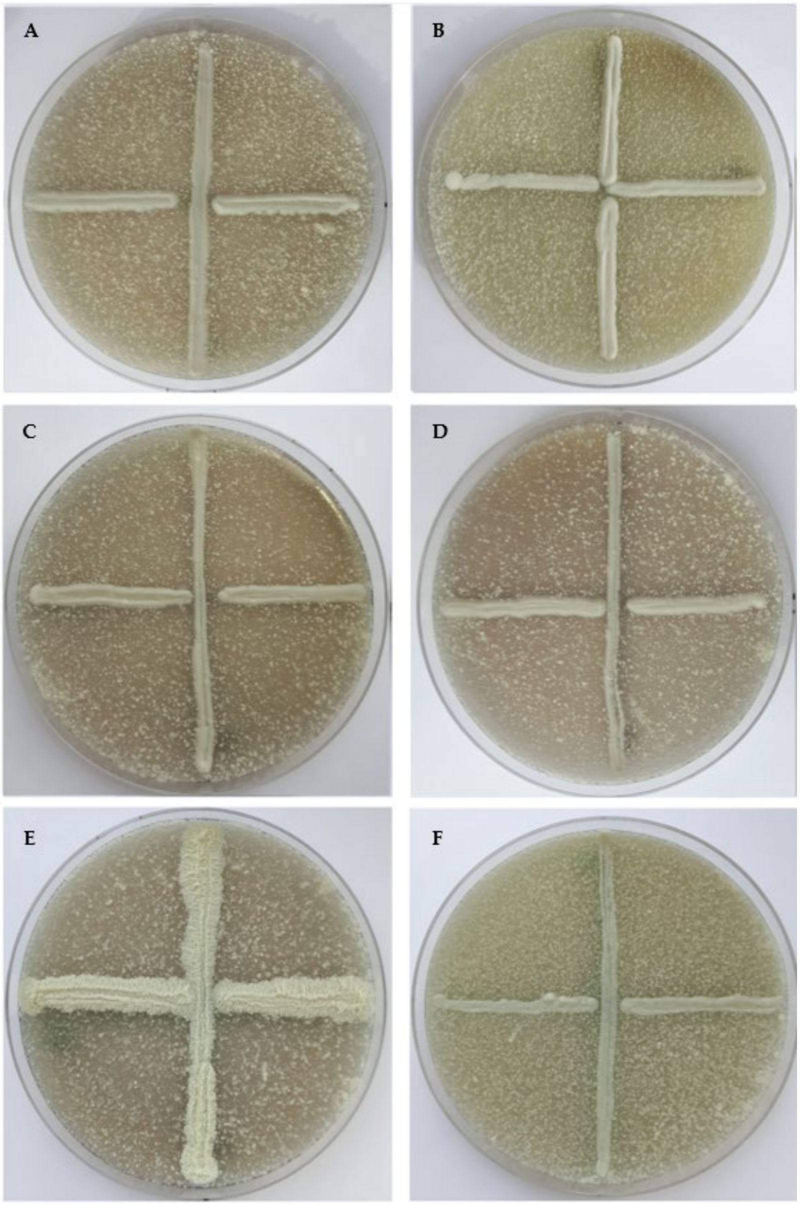

Figure S6. Killer activity test of *D. hansenii* strains before (horizontal streaks) and after (vertical streaks) curing of VLEs against sensitive *Y. lipolytica* PII6a yeast strain (in lawn) on YPG agar pH 7.0 with 2.5% NaCl at 14°C. (A) 4e, (B) 5c, (C) 7g, (D) 8h, (E) CBS 7848, (F) CBS 770.

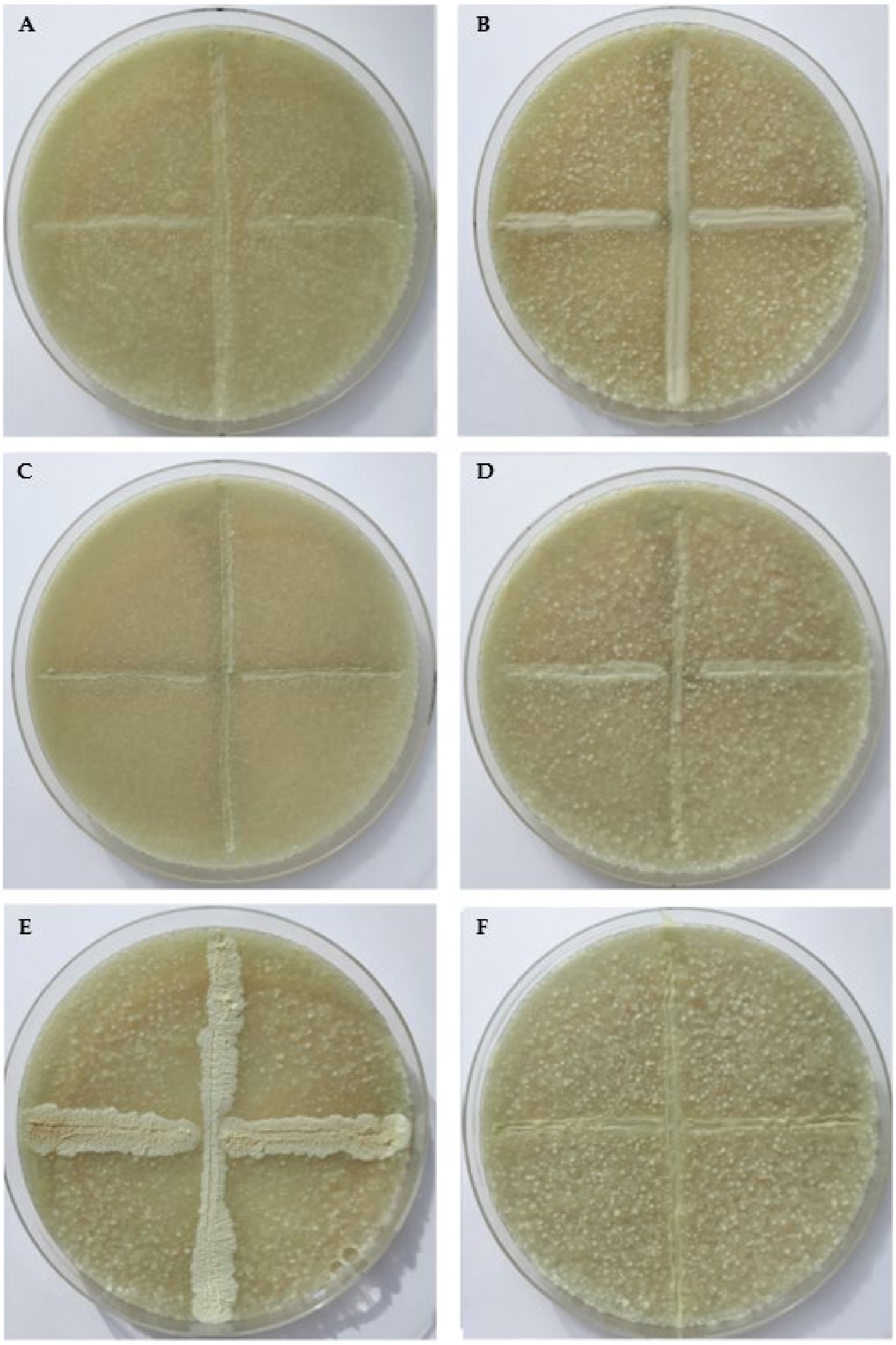

Figure S7. Killer activity test of *D. hansenii* strains before (horizontal streaks) and after (vertical streaks) curing of VLEs against sensitive *Y. lipolytica* PII6a yeast strain (in lawn) on YPG agar with 2.5% NaCl pH 7.0 at 28 °C. (**A**) 4e, (B) 5c, (C) 7g, (D) 8h, (E) CBS 7848, (F) CBS 770.

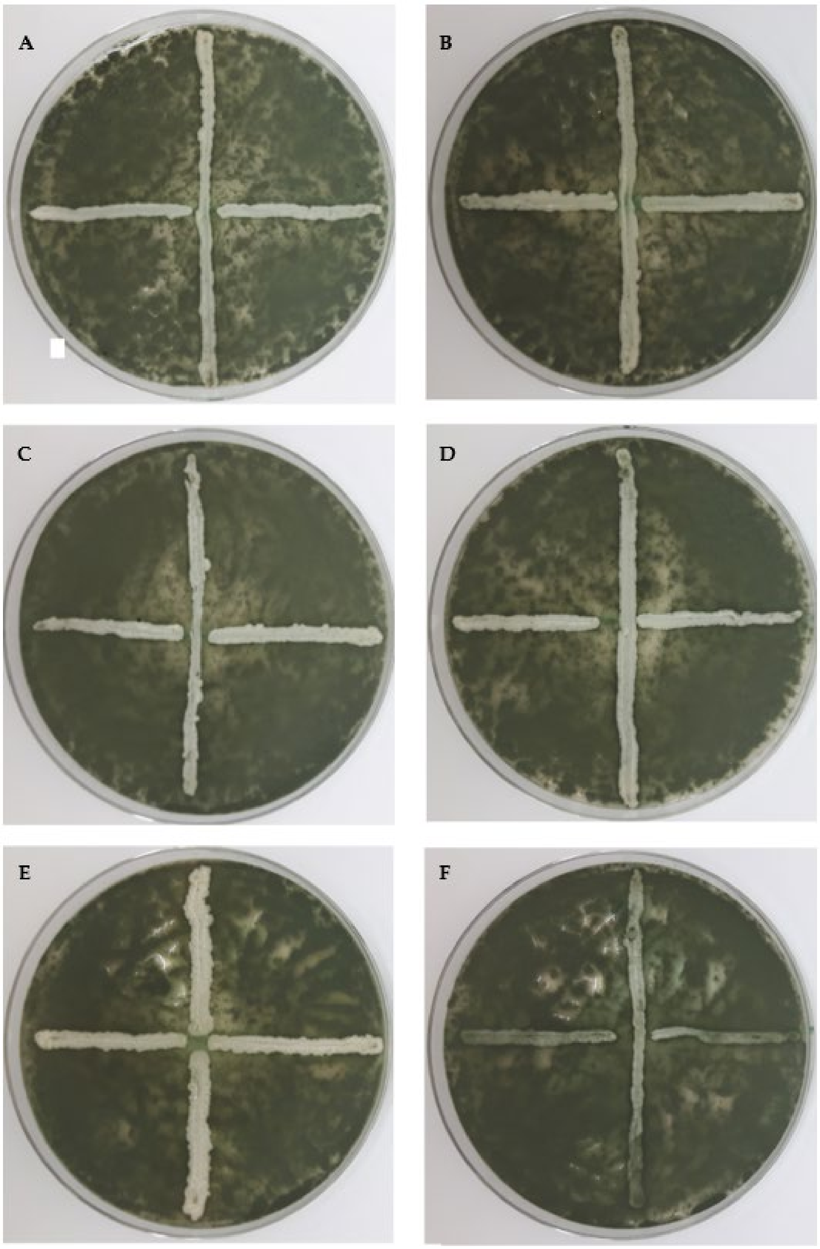

Figure S8. Killer activity test of *D. hansenii* strains before (horizontal streaks) and after (vertical streaks) curing of VLEs against sensitive *P. roqueforti* PR1 (in lawn) on PDA with 2.5% NaCl pH 7.0 at 14°C. (A) 4e, (B) 5c, (C) 7g, (D) 8h, (E) CBS 7848, (F) CBS 770.

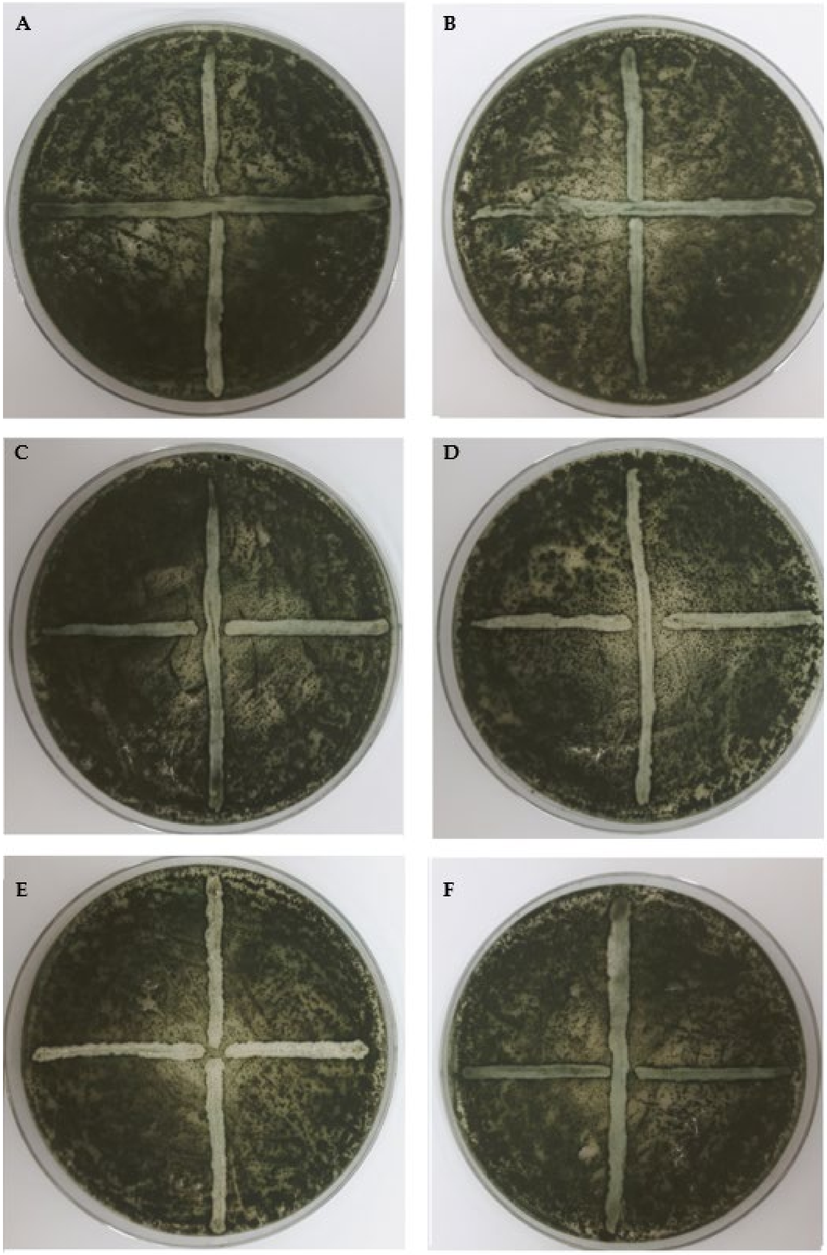

Figure S9. Killer activity test of *D. hansenii* strains before (horizontal streaks) and after (vertical streaks) curing of VLEs against sensitive *P. roqueforti* PR1 (in lawn) on PDA with 2.5% NaCl pH 7.0 at 28°C. (A) 4e, (B) 5c, (C) 7g, (D) 8h, (E) CBS 7848, (F) CBS 770.

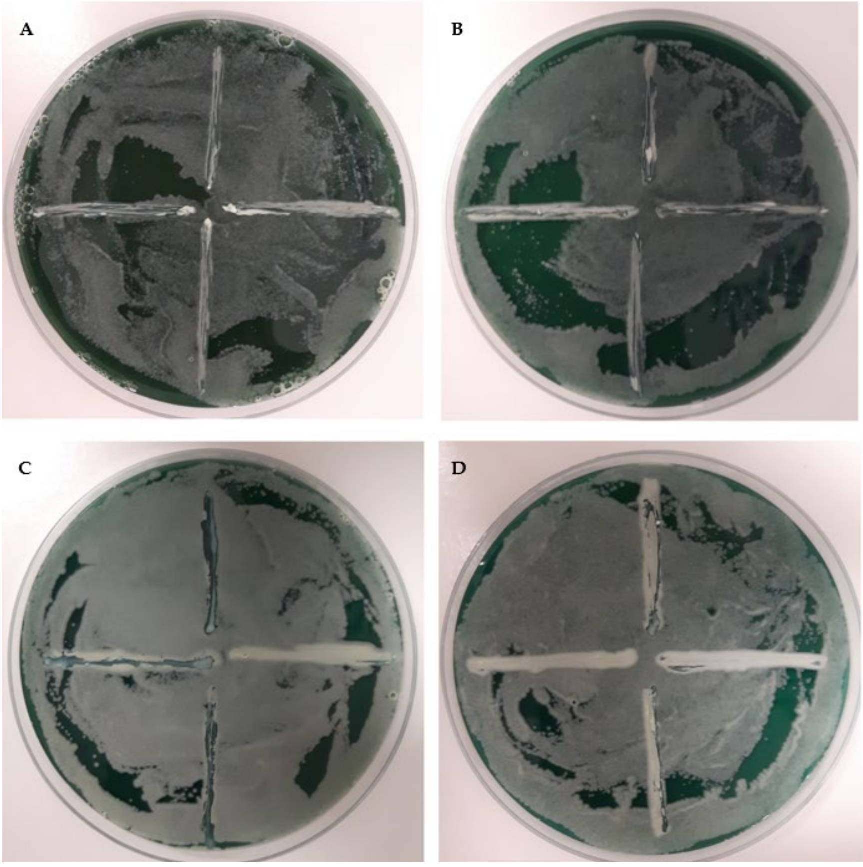

Figure S10. Killer activity test of *D. hansenii* strains before (streaks) and after (in lawn) curing of VLEs on YMB pH 7.0 with 2.5% NaCl at 14 °C. (A) 4e, (B) 5c, (C) 7g, (D) 8h. The same results were obtained on YMB pH 4.6 with 2.5% NaCl at 14 °C.

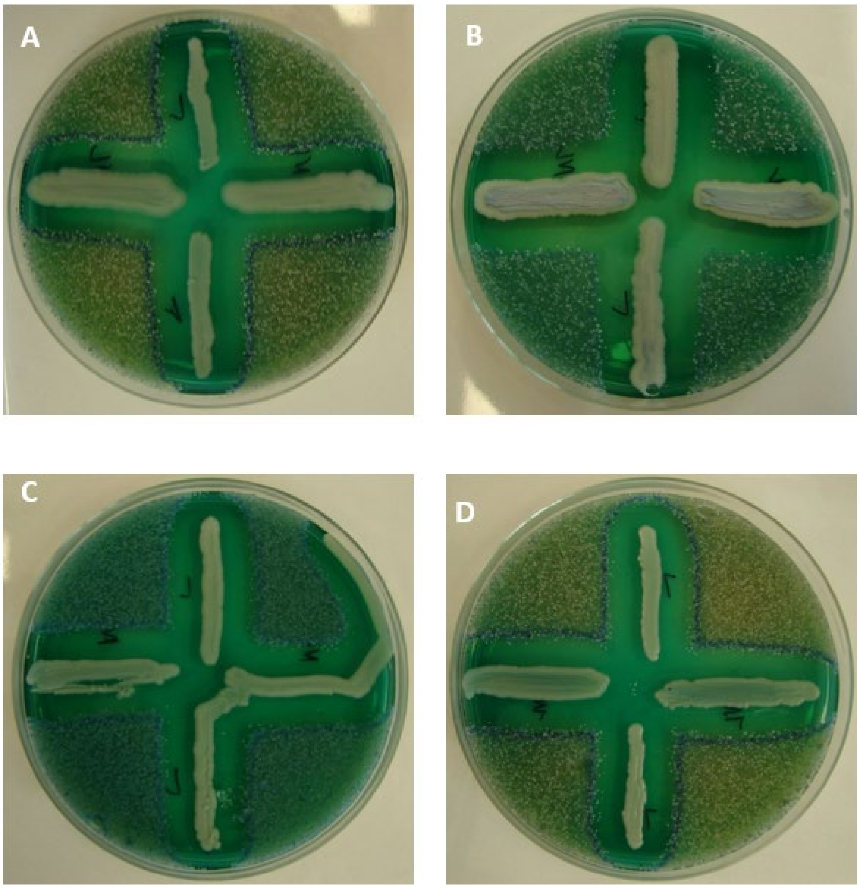

Figure S11. Killer activity of *D. hansenii* strains before (horizontal streaks) and after (vertical streaks) curing of VLEs against sensitive *Y. lipolytica* PII6a yeast strain (in lawn, dead cells colored in blue) on YMB pH 4.6 with 2.5% NaCl at 14°C for 72h. (A) 4e, (B) 5c, (C) 7g, (D) 8h.

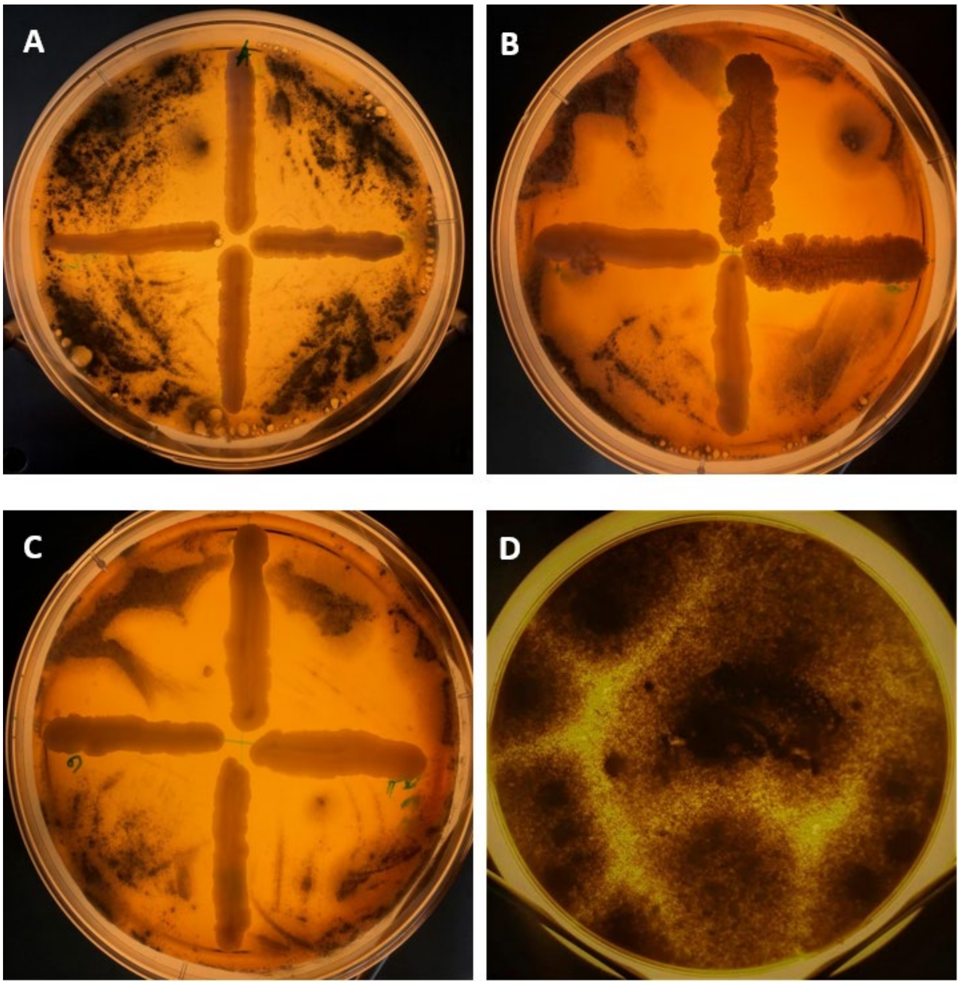

Figure S12. Antifungal activity of *D. hansenii* strains before (vertical streaks) and after (horizontal streaks) curing of linear plasmids against sensitive *P. roqueforti* PR1 (in lawn) on PDA + 2.5% NaCl pH 4.6 at 14°C. (A) 4e left and up, 5c down and right, (B) 7g left and down, CBS 7848 up and right, (C) 8h left and up, CBS 770, (D) control plate.

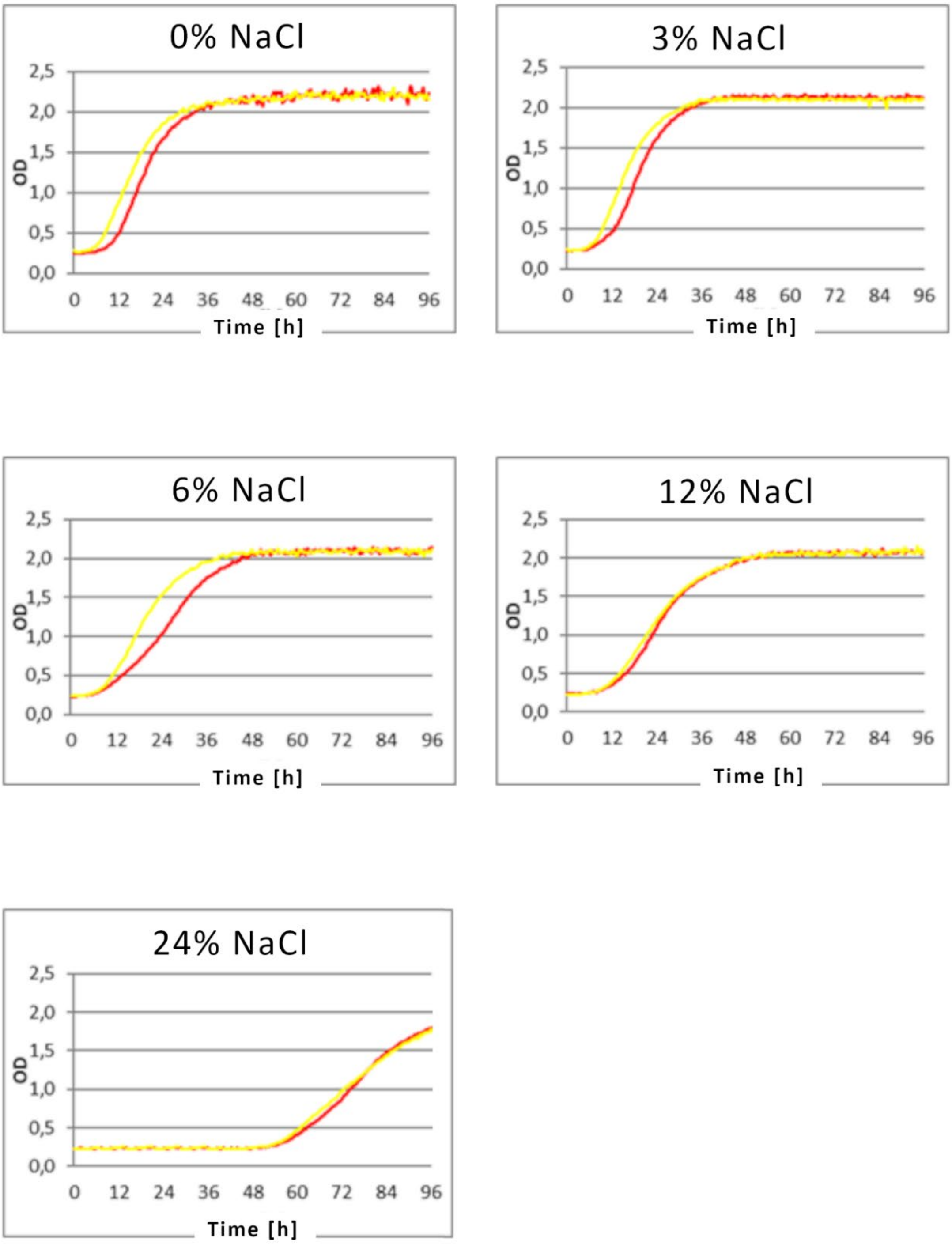

Figure S13. The growth of *D. hansenii* 5c strain before (red line) and after (yellow line) VLEs curing in YPD medium with different NaCl concentration. Cultures performed in BioscreenC plotting OD value using wide band filter of range 420-580 nm.
